# Investigation into the Novel Microalgae Membrane Bioreactor with Internal Circulating Fluidized Bed for Marine Aquaculture Wastewater Treatment

**DOI:** 10.3390/membranes10110353

**Published:** 2020-11-18

**Authors:** Yi Ding, Zhansheng Guo, Junxue Mei, Zhenlin Liang, Zhipeng Li, Xuguang Hou

**Affiliations:** 1 Marine College, Shandong University, Weihai 264209, China; dingyi@sdu.edu.cn (Y.D.); guozhansheng@sdu.edu.cn (Z.G.); meijunxue@sdu.edu.cn (J.M.); liangzhenlin@sdu.edu.cn (Z.L.); 2 State Key Laboratory of Urban Water Resources and Water Environment, School of Marine Science and Technology, Harbin Institute of Technology at Weihai, Weihai 264200, China

**Keywords:** microalgae, membrane bioreactor, internal circulating fluidized bed, marine aquaculture wastewater, nitrogen and phosphorus removal

## Abstract

A microalgae membrane bioreactor (MMBR) with internal circulating fluidized bed (ICFB) was constructed at room temperature to study the removal efficiency of marine aquaculture wastewater pollutants and continuously monitor the biomass of microalgae. Within 40 days of operation, the removal efficiency of NO_3_^−^–N and NH_4_^+^–N in the ICFB-MMBR reached 52% and 85%, respectively, and the removal amount of total nitrogen (TN) reached 16.2 mg/(L·d). In addition, the reactor demonstrated a strong phosphorus removal capacity. The removal efficiency of PO_4_^3−^–P reached 80%. With the strengthening of internal circulation, the microalgae could be distributed evenly and enriched quickly. The maximum growth rate and biomass concentration reached 60 mg/(L·d) and 1.4 g/L, respectively. The harvesting of microalgae did not significantly affect the nitrogen and phosphorus removal efficiency of ICFB-MMBR. The membrane fouling of the reactor was investigated by monitoring transmembrane pressure difference (TMP). Overall, the membrane fouling cycle of ICFB-MMBR system was more than 40 days.

## 1. Introduction

With the industrialization and intensive development of aquaculture, the density and scale of aquaculture are expanding, and the water demand for aquaculture is also increasing. However, a large amount of aquaculture wastewater will be produced in the process of aquaculture [1]. The wastewater mainly contains inorganic nitrogen, phosphorus, organic pollutants and other pollutants [2]. If directly discharged into the water, it will lead to eutrophication. In addition, ammonia and nitrite also have strong toxic effects on aquatic organisms. These above problems have restricted the sustainable development of marine aquaculture. In order to remove the pollutants in wastewater, physical and chemical methods can obtain a high nutrient removal efficiency, but there are some shortcomings such as high energy consumption and secondary pollution [3]. In contrast, biochemical methods have the advantages of low cost, low energy consumption and low secondary pollution. Therefore, biochemical methods are favored by many researchers. At present, many kinds of nitrogen and phosphorus removal processes have been reported, but there will be problems such as high sludge yield, high-energy consumption of aeration and poor phosphorus removal efficiency.

Microalgae, as a kind of bioenergy, can effectively absorb nitrogen and phosphorus in the environment during the growth process [4,5]. Meanwhile, microalgae can synthesize carbohydrates from light energy, and produce proteins, oils and other substances through further biochemical reactions [6]. Thus, microalgae are widely used in aquaculture, food, biodiesel, agricultural fertilizer and other fields [7,8,9,10]. At present, there is much research on the removal of nitrogen and phosphorus in wastewater by microalgae. However, most of the research objects are mainly aimed at low salinity wastewater [11,12]. On the other hand, most of these studies are mainly based on sequencing batch test. Microalgae metabolites cannot be excreted in the sequencing batch reactor, which affects the nitrogen and phosphorus removal efficiency of microalgae reactor. Membrane bioreactor (MBR) has been considered to be one of the most effective technologies to treat urban and industrial wastewater in the world. There are many advantages of MBR, such as good effluent quality, high organic load, low sludge yield and small occupation area [13]. For this reason, MBR has been widely used in wastewater treatment field. In addition, the pollutants could be degraded efficiently in MBR, and the biomass could be effectively retained inside the reactor. Therefore, the combination of microalgae and MBR process can be considered.

Recently, some researchers have used microalgae membrane bioreactor to treat domestic sewage and aquaculture wastewater [14,15], and achieved remarkable removal efficiency. However, most microalgae membrane bioreactors have a single structure and poor fluidity of algal fluid in the reactor [16]. Enhanced flow of microalgae in the reactor cannot only improve the efficiency of mass transfer, but also effectively alleviate the precipitation of microalgae. Therefore, based on the absorption of nitrogen and phosphorus by microalgae, a novel microalgae membrane bioreactor (MMBR) with internal circulating fluidized bed (ICFB) was constructed to efficiently degrade pollutants and enrich microalgae in order to realize the reuse of marine aquaculture wastewater. At the same time, the membrane fouling was continuously monitored in the reactor, which would provide a reference for the treatment of marine aquaculture wastewater and the utilization of microalgae.

## 2. Materials and Methods

### 2.1. Membrane Bioreactor Setup

**Figure 1:**
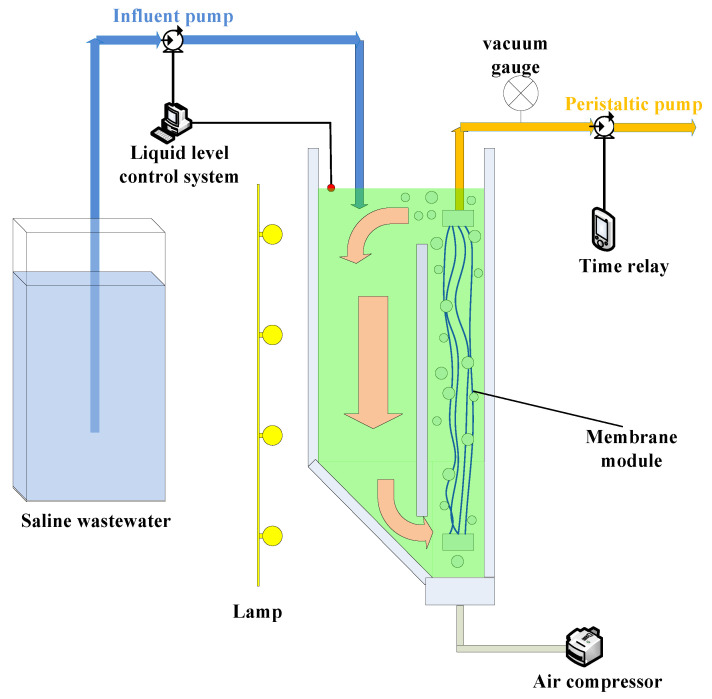
The schematic representation of the microalgae membrane bioreactor (MMBR) with internal circulating fluidized bed (ICFB).

In this experiment, the ICFB-MMBR was made of plexiglass with a working volume of 3 L, as shown in Figure 1. The polyvinylidene fluoride (PVDF) hollow-fiber ultrafiltration membrane (Motian Co. Ltd., Tianjin, China) was installed in the aeration zone. The pore size and surface area of the membrane was 0.03 μm and 0.02 m^2^, respectively. Local aeration and central baffle were used to circulate microalgae liquid in the reactor and slow down the precipitation of microalgae. The air flow rate was 0.8 L/min during the experiment. Meanwhile, the surface of membrane was scoured via the shear stress of bubble. The liquid level of the reactor was maintained by the control system. The effluent was filtered out from the membrane module by peristaltic pump. By adjusting the speed of the peristaltic pump, the flow rate of effluent was controlled. The membrane module was operated at a constant flux with a filtration cycle of 8 min on and 2 min off via the time relay to alleviate membrane fouling. The hydraulic retention times (HRT) was kept at 1 day with the room temperature and 12 h light–12 h dark cycle. Transmembrane pressure (TMP) was recorded through vacuum gauge. The TMP data presented were based on the measurements conducted after the MMBR reached steady-state. The steady-state, herein, referred to the experimental period approximately after 20 cycle periods. Only the steady-state results were reported in the paper. Once the TMP reached 30 kPa in the MMBR, the membrane modules were taken out and cleaned. The membrane foulants were carefully scraped off from the membrane surface. The membrane modules were reloaded into the bioreactors to run after cleaning.

The fluorescent lamp was placed around the reactor with a total illumination intensity of about 4000 lx. The influent pH remained at about 7 by adding 1 mol/L HCl and 1 mol/L NaOH.

### 2.2. Microalgae and Cultivation

In this experiment, *Platymonas helgolandica tsingtaoensis* from the Institute of Oceanography, Chinese Academy of Sciences was used as biogenic source. The microalgae cells were cultured in light incubator with f/2 medium. The flasks were shaken 3 times a day to prevent precipitation.

### 2.3. Marine Aquaculture Wastewater

Seawater collected from Huang Hai (Shidao, Weihai, China) was used to synthetize the saline wastewater. The main pollutants in wastewater were as follows: 30 mg/L NaNO_3_, 55 mg/L NH_4_Cl, 17 mg/L KH_2_PO_4_. No buffering agent such as NaHCO_3_ was added. In addition, a proper amount of trace elements was added according to the composition of f/2 medium.

### 2.4. Treatment Performance and Membrane Fouling

The influent and effluent samples of the reactor were collected every two days. After filtration with 0.45 μm filter membrane, the ammonia (NH_4_^+^–N), nitrate (NO_3_^−^–N), nitrite (NO_2_^−^–N), phosphate (PO_4_^3−^–P), total nitrogen (TN) and pH value of the samples were determined. Meanwhile, the microalgae biomass and membrane fouling of the reactor were also investigated. TMP was determined directly via precise vacuum pressure. The extracellular polymeric substance (EPS) extracted from the mixed liquor samples was also analyzed.

### 2.5. Analytical Methods

Measurement of ammonia nitrogen (NH_4_^+^–N), nitrate nitrogen (NO_3_^−^–N), nitrite nitrogen (NO_2_^−^–N) and phosphorus (PO_4_^3−^–P) were carried out using an ultraviolet-visible spectrophotometer (UV-1800, Shimadzu, Kyoto, Japan) according to the standard methods [17]. The biomass of microalgae was determined via optical density (OD) method [18]. The EPS consists of soluble EPS (S-EPS) and bound EPS (B-EPS). The composition of EPS was analyzed in terms of protein and carbohydrate. Proteins were analyzed by the Lowry method [19] and carbohydrates were assessed using the phenol-sulfuric method [20], respectively. The pH value was measured by a pH meter (FE20K, Mettler Toledo, Switzerland). Nutrient removal efficiency and removal amount were calculated by the following two formulas:

Nutrient removal efficiency = (1—effluent concentration/influent concentration) × 100%

Nutrient removal amount = (influent concentration − effluent concentration)/HRT

## 3. Results and Discussion

### 3.1. Microalgae Growth in the ICFB-MMBR

**Figure 2:**
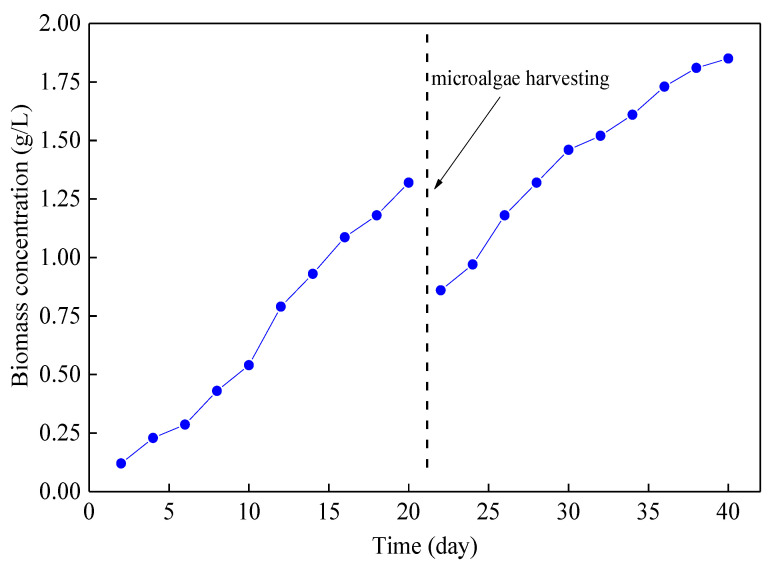
Microalgae growth in the microalgae membrane bioreactor (MMBR) with internal circulating fluidized bed (ICFB).

During the 40 days’ operation of the reactor, the growth of microalgae in the reactor was continuously monitored, and the results are shown in Figure 2. The initial biomass was 0.12 g/L. The biomass concentration of microalgae reached 1.32 g/L on the 20th day, and the average growth rate was 60.0 mg/(L·d). In order to realize the resource utilization of microalgae and not affect the pollutant treatment level of the reactor excessively, the algal liquid was collected in the reactor. From the 20th day, the biomass concentration of microalgae increased from 0.86 g/L to 1.85 g/L (the 40th day), and the average growth rate was 49.5 mg/(L·d).

### 3.2. Pollutant Removal in the ICFB-MMBR

**Figure 3:**
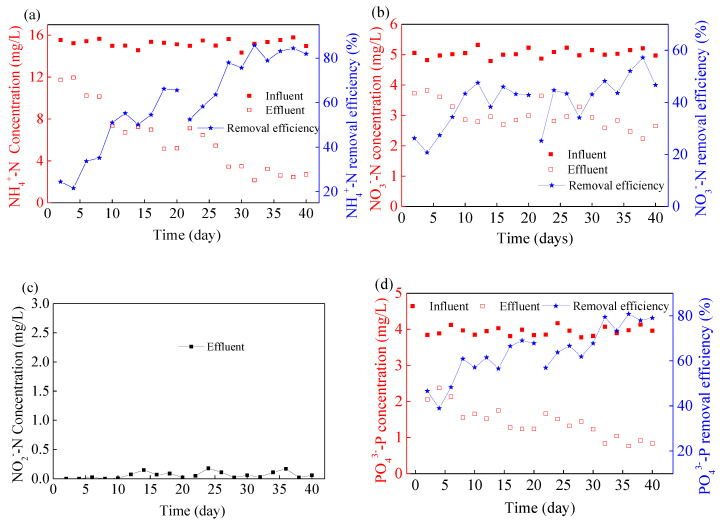
The removal of NH_4_^+^–N (**a**), NO_3_^−^–N (**b**), NO_2_^−^–N (**c**) and PO_4_^3−^–P (**d**) in the internal circulating fluidized bed (ICFB)—microalgae membrane bioreactor (MMBR).

The removal efficiency of pollutants was monitored with mariculture wastewater as influent during 40 days’ operation of the reactor. The removal efficiency of NH_4_^+^–N was about 25%, the concentration of NH_4_^+^–N in effluent was 11.7 mg/L, and the quality of effluent is poor, as shown in Figure 3a. With the growth of microalgae biomass, the effluent NH_4_^+^-N concentration decreased to 7 mg/L (on the 10th day). The removal efficiency NH_4_^+^–N was stable at 65% and microalgae biomass reached 1.32 g/L (on the 20th day). Some studies have reported that longer retention time of microalgae will lead to a decrease in TN removal efficiency and reduce the productivity of microalgae biomass [21]. In order to achieve better utilization of microalgae and not affect the efficiency of nitrogen and phosphorus removal efficiency due to the low biomass in the reactor, 1 L microalgae liquid in the reactor was harvested on the 20th day. After microalgae harvesting, the biomass of microalgae in the reactor decreased to about 0.86 g/L, and the NH_4_^+^–N concentration in effluent increased slightly. However, the effluent NH_4_^+^–N concentration decreased to 5.4 mg/L on the 26th day, and the growth rate of microalgae biomass was consistent with that of the previous stage. In the following period, with the increase of biomass, the concentration of NH_4_^+^–N in effluent was stable at about 2.5 mg/L, and the removal efficiency of NH_4_^+^–N reached about 84%. On the 36th day, the growth of biomass tended to be slow, and the maximum biomass 1.85 g/L was reached on the 40th day. In addition, the flow pattern of microalgae in the reactor was more uniform during the whole operation period. Except for a small amount of microalgae on the inner wall of the reactor, there was no obvious precipitation, which indicated that MMBR could provide a good environment for the growth of microalgae.

During the 40 days’ operation of MMBR, the removal efficiency of NO_3_^-^–N was 26–55%, as shown in Figure 3b. By comparing the removal efficiency of NO_3_^-^–N and NH_4_^+^–N, it is known that the removal efficiency of NH_4_^+^–N is better. It had been reported that when NH_4_^+^–N and NO_3_^-^–N coexisted, microalgae preferentially utilized NH_4_^+^–N, and the presence of NH_4_^+^–N inhibited the absorption of NO_3_^−^–N by algae cells [22]. Similar experimental phenomena have been reported, which may be the main reason for the low removal efficiency of NO_3_^−^–N in this experiment.

Nitrite is the intermediate product of many biochemical reactions of microorganisms, but excessive accumulation of NO_2_^−^–N will cause toxic effects on microorganisms and inhibit biochemical reactions, which is not conducive to the growth of microalgae and the stable operation of the reactor. Thus, the concentration of NO_2_^−^–N was also monitored during the operation of the reactor. The results showed that low concentration of NO_2_^−^–N was detected in the effluent in a few days, as shown in Figure 3c. Overall, there was no obvious accumulation of NO_2_^−^-N during 40 days’ operation. Most studies have shown that nitrate reductase and nitrite reductase successively transformed NO_3_^−^–N and NO_2_^−^–N to NH_4_^+^–N in algae cells, and finally assimilated to amino acids in the form of NH_4_^+^–N. Therefore, NO_2_^−^–N in effluent may be due to the transformation of NO_3_^−^–N. Praveen and Loh (2016) constructed a microalgae photobioreactor to treat tertiary effluent, and no NO_2_^−^-N was accumulated under HRT of 4 days [23]. Therefore, the longer HRT may help to alleviate the accumulation of NO_2_^−^–N.

Compared with other microorganisms, microalgae are more likely to absorb phosphorus in wastewater to increase biomass. The PO_4_^3-^–P concentration in effluent was about 2.3 mg/L during the first 6 days of operation, as shown in Figure 3d. From the 16th to the 20th day, the PO_4_^3−^–P concentration in effluent could be maintained at 1.2 mg/L. After harvesting microalgae on the 20th day, the PO_4_^3−^–P concentration in the effluent increased slightly, which was similar to that of NH_4_^+^–N. Thereafter, with the increase of microalgae biomass, the concentration of PO_4_^3−^–P in effluent was stable below 0.9 mg/L with a high removal efficiency.

Praveen and Loh (2016) reported that in the continuous microalgae-membrane bioreactor to treat tertiary effluent, the removal amount of TN and TP were 3.6 mg/(L·d) and 0.8 mg/(L·d), respectively [23]. Gao et al. (2016) showed that in the continuous streamer-membrane bioreactor, the growth rate of microalgae was 42.6 mg/(L·d), and the removal amount of TN and TP was 5.85 mg/(L·d) and 0.42 mg/(L·d), respectively [24]. In this experiment, through continuous monitoring of water quality, the TN and TP removal amount of the reactor was investigated. With the increase of microalgae biomass, the removal amount of TN and TP could reach 13–16.2 mg/(L·d) and 2.3–3.2 mg/(L·d), respectively. The average growth rate of microalgae was 55 mg/(L·d). The removal efficiency of pollutants and the growth rate of microalgae are higher than those reported above. This may be due to the uniform distribution of microalgae in MMBR, which can significantly improve the efficiency of mass transfer and biochemical reaction of microalgae. During the operation of the reactor, the maximum biomass of microalgae was 1.85 g/L, and the average growth rates of microalgae before and after harvesting were 60 mg/(L·d) and 49.5 mg/(L·d), respectively. The reason for this difference may be that with the increase of biomass concentration, limited light intensity will become a limiting factor for the growth of microalgae [25]. During the later stage of operation, more algal adhesion was observed in the inner wall of the reactor, which might reduce the light intensity to some extent. As a result, the growth rate of microalgae slowed down in the last few days. Overall, the growth rate of microalgae was at a higher level in the current study.

### 3.3. Variations of pH in the ICFB-MMBR

**Figure 4:**
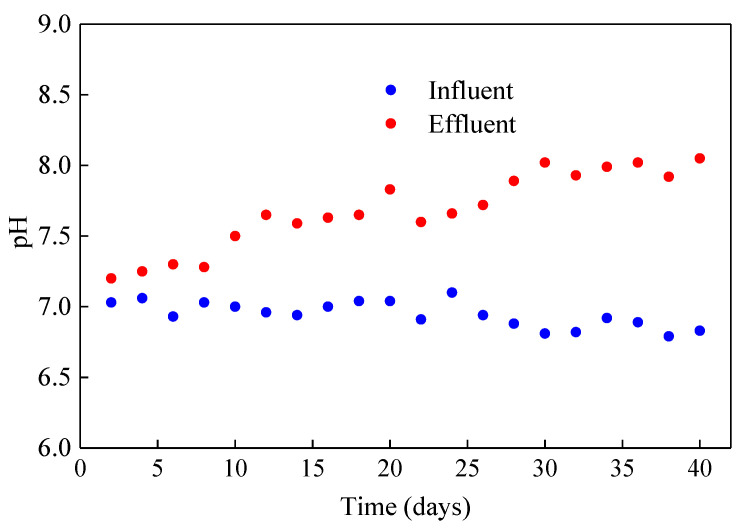
Variations of pH in the influent (blue) and effluent (red) of the internal circulating fluidized bed (ICFB)—microalgae membrane bioreactor (MMBR).

During the cultivation of microalgae, there are many factors affecting the growth of microalgae, among which pH is one of the most important environmental factors in microalgae culture, because pH determines the solubility and mass transfer efficiency of carbon dioxide and matrix, and has a significant impact on the metabolism of microalgae [26]. When the pH of wastewater is lower than 8, it is more conducive to the formation of CO_2_, which was easy to be immobilized by microalgae [27]. In this experiment, the influent pH was stabilized at about 7 by adding acid and alkali, and the effluent pH was between 7.2 and 8, as shown in Figure 4. Within this pH range, chemical processes such as volatilization and precipitation have little effect on ammonia and phosphate removal. Thus, it can be inferred that nitrogen and phosphorus removal mainly depends on the growth of microalgae biomass.

Many studies showed that that CO_2_ fixation would lead to a gradual pH increase, but excessive pH would affect the growth of algae cells. Furthermore, ammonia nitrogen is more toxic to microalgae under the condition of high pH [28]. During the microalgae wastewater treatment, many researchers adjust the pH value by adding CO_2_ to the system. However, overuse of carbon dioxide will increase operating costs. In this experiment, aeration (air) equipment was used without additional CO_2_ supply, and the effluent pH could be controlled below 8 without a buffer. Meanwhile, the effluent pH tended to increase with the improvement of nitrogen and phosphorus removal efficiency of the reactor, which showed that ICFB-MMBR could create a good environment for the growth of microalgae under the operating parameters.

### 3.4. Variations of TMP in the ICFB-MMBR

**Figure 5:**
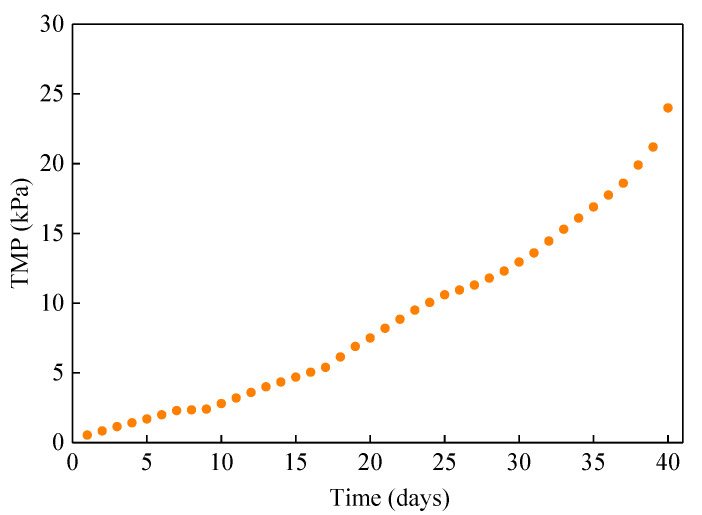
The variations in TMP throughout the experimental period.

TMP can directly reflect membrane fouling of the reactor. Therefore, TMP was continuously monitored within 40 days of reactor operation, during which no physical cleaning of membrane module was carried out. It can be seen that the variation of TMP almost increased at the constant rate, as shown in Figure 5. In the first 7 days, TMP rose rapidly from 0.5 kPa to 2.3 kPa. However, the TMP rising speed slowed down on the 8th day. At the same time, microalgae attachment was observed on the surface membrane module. Subsequently, the TMP rising speed began to increase on the 36th day. Meanwhile, it was observed that there were much more microalgae attached to the membrane surface, which indicated that the membrane pore blockage was more serious. Finally, the TMP increased to 24 kPa on the 40th day. Overall, the membrane fouling was not severe in the reactor operation. This had also been reported, that the membrane fouling of microalgae system was lighter than that of activated sludge MBR [29].

### 3.5. Variations of EPSs in the ICFB-MMBR

**Figure 6:**
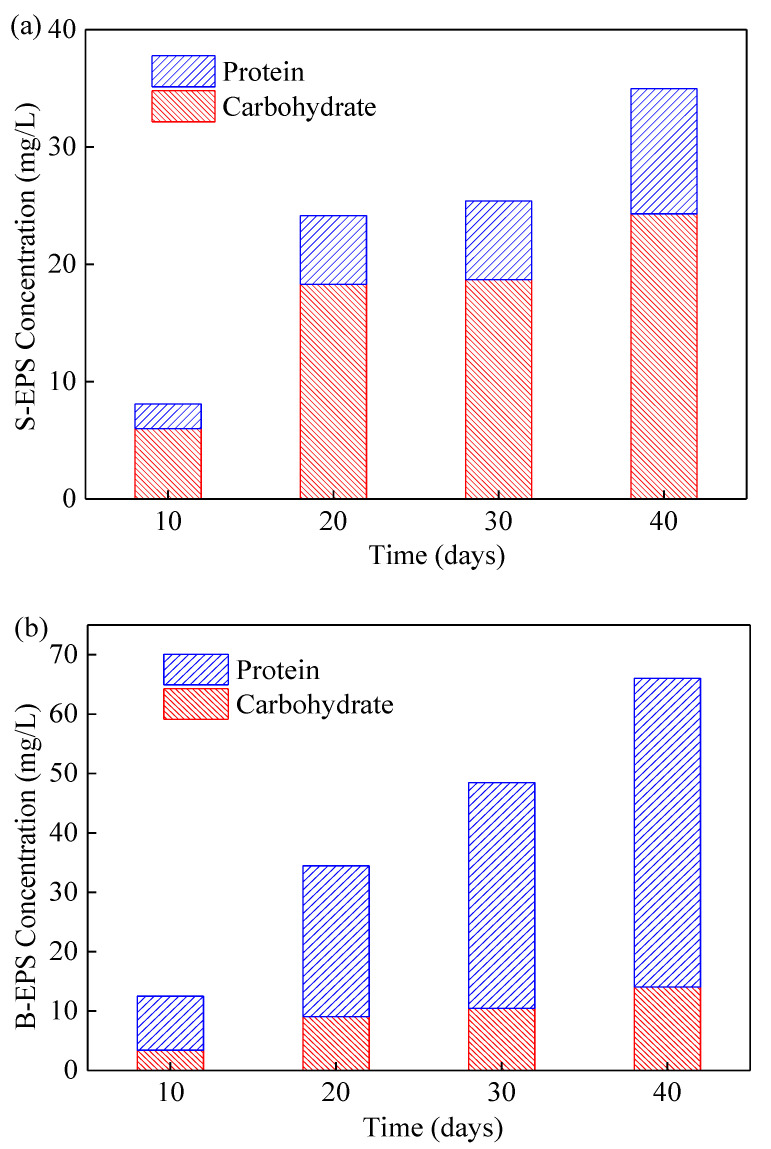
Concentrations of S-EPS (**a**) and B-EPS (**b**) in the internal circulating fluidized bed (ICFB)—microalgae membrane bioreactor (MMBR).

EPS is closely related to the physiological state of microorganisms and plays an important role in the formation of membrane fouling. Carbohydrate and protein are known to be the main components of EPSs. Therefore, the EPSs content was monitored during the operation of the reactor, as shown in Figure 6. As for S-EPS, carbohydrate accounted for 70–75% of S-EPS content compared with protein (25–30%). Because carbohydrate is one of the main parts of the algae cells wall, it would be released into the mixed liquor when the microalgae multiply [30]. In contrast, protein was the main component of B-EPS, as shown in Figure 6b. Since N is an essential constituting element of proteins, higher N removal efficiency observed in this experiment might result in production of large amounts of protein in B-EPS. Additionally, EPS and cells were co-deposited during membrane filtration, forming the cake layer formation, which could alleviate membrane fouling to a certain extent [31].

In general, the contents of S-EPS and B-EPS increased significantly with the growth of microalgae biomass. However, the increase of EPS will lead to serious membrane fouling. Therefore, the proper harvesting of microalgae is of great significance to alleviate membrane fouling.

## 4. Conclusions

In this study, a microalgae membrane bioreactor (MMBR) with internal circulating fluidized bed (ICFB) was constructed to treat high salinity wastewater. The operation results showed that ICFB-MMBR could achieve high removal efficiency of nitrogen and phosphorus, especially for ammonia nitrogen and phosphate in wastewater. Moreover, microalgae could be enriched quickly in the reactor. Meanwhile, the removal capacity of the reactor was not significantly affected by the harvesting of microalgae on the 20th day. The results of TMP variations showed that the membrane fouling of ICFB-MMBR system was relatively light.
